# Supplementary figures and images for: Reinvestigating the phylogeny of Myriapoda with more extensive taxon sampling and novel genetic perspective
Source: PeerJ. 2021 Dec 23;9:e12691. doi: 10.7717/peerj.12691 (PMC8710254; doi:10.7717/peerj.12691)

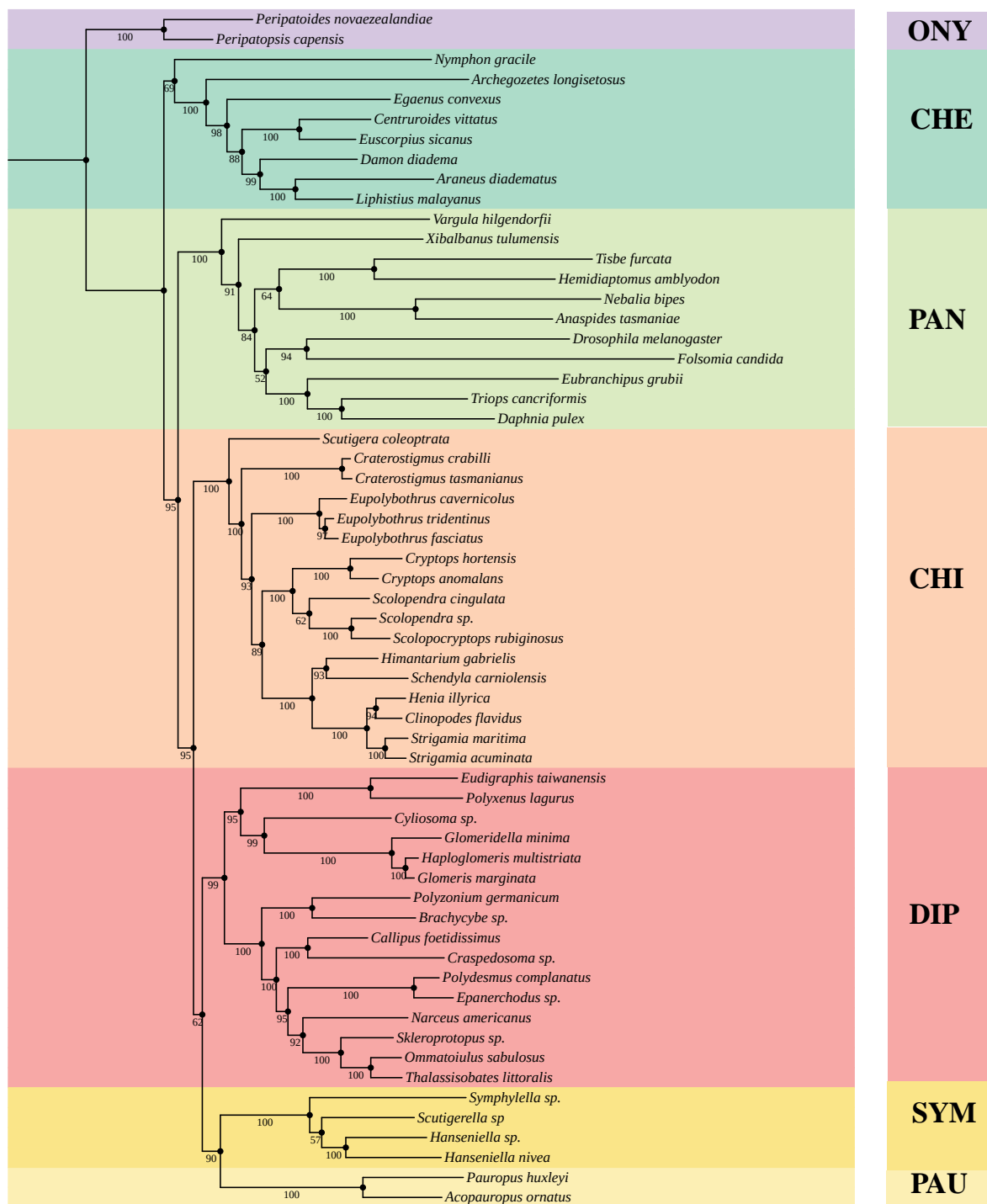

Tree scale: 0.1

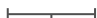

Supplement: Supplemental Information 3 [file peerj-09-12691-s003.pdf]

Tree scale: 0.1

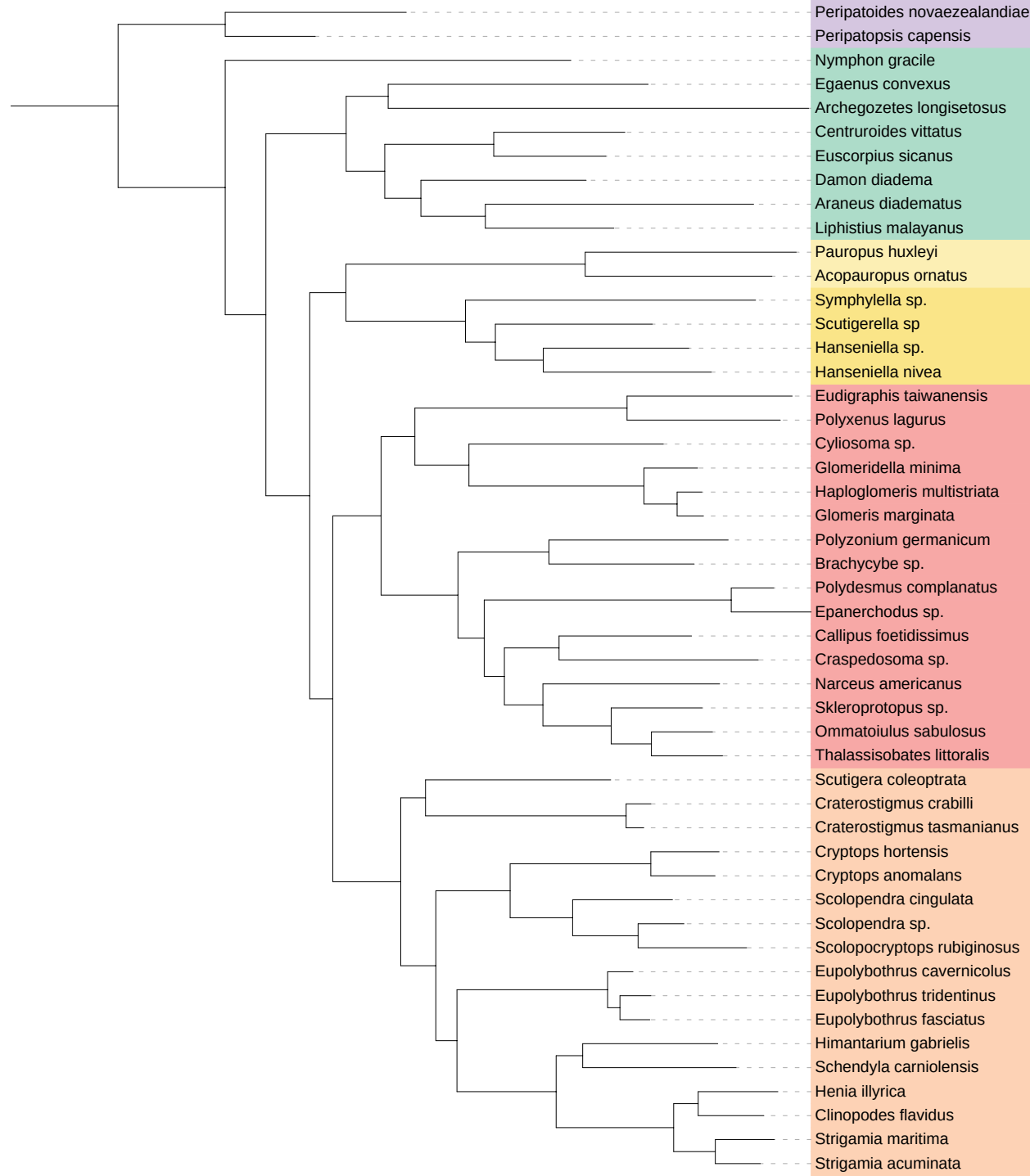

Supplement: Supplemental Information 4 [file peerj-09-12691-s004.pdf]

Tree scale: 0.1

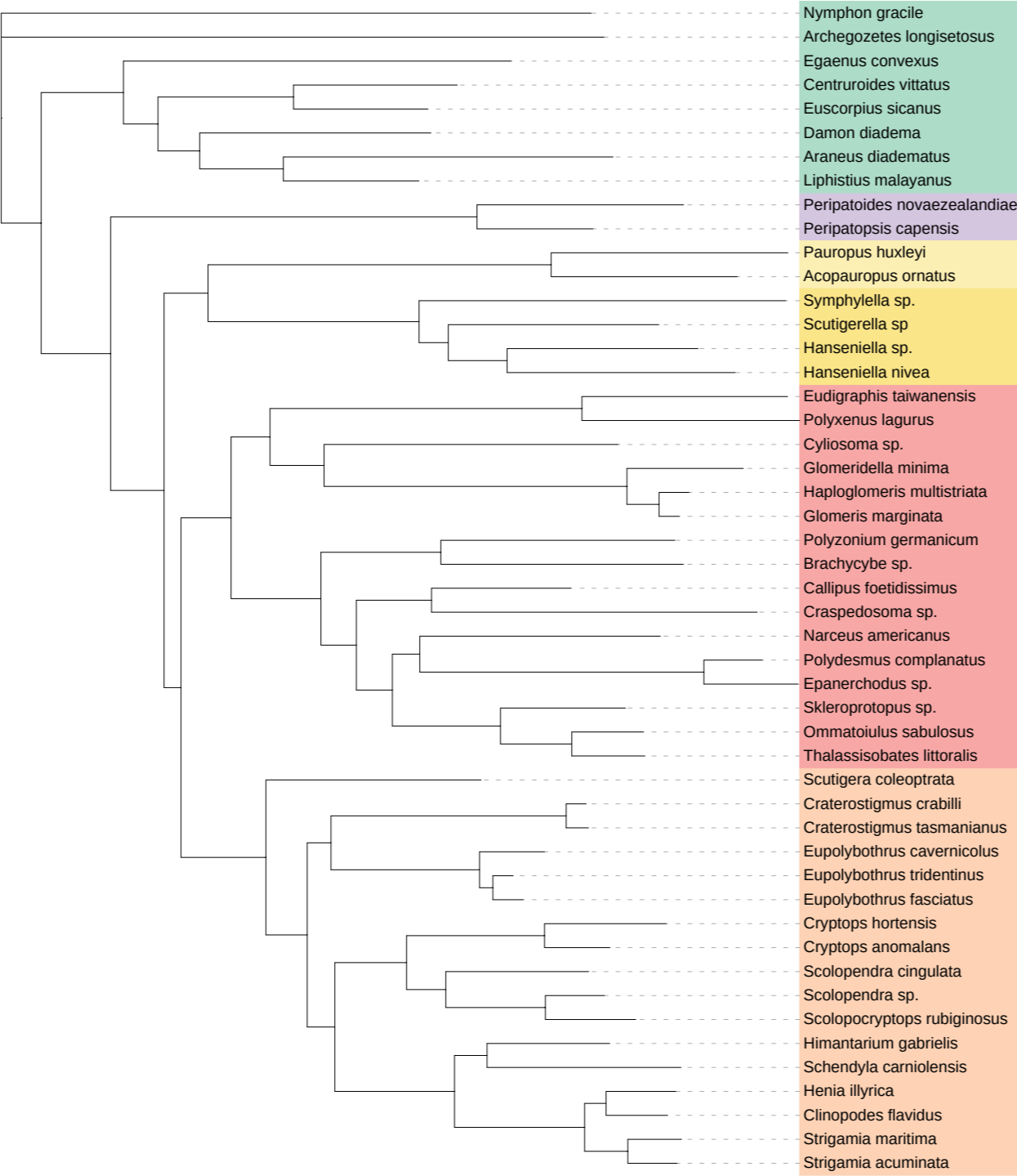

Supplement: Supplemental Information 5 [file peerj-09-12691-s005.pdf]

Tree scale: 0.1

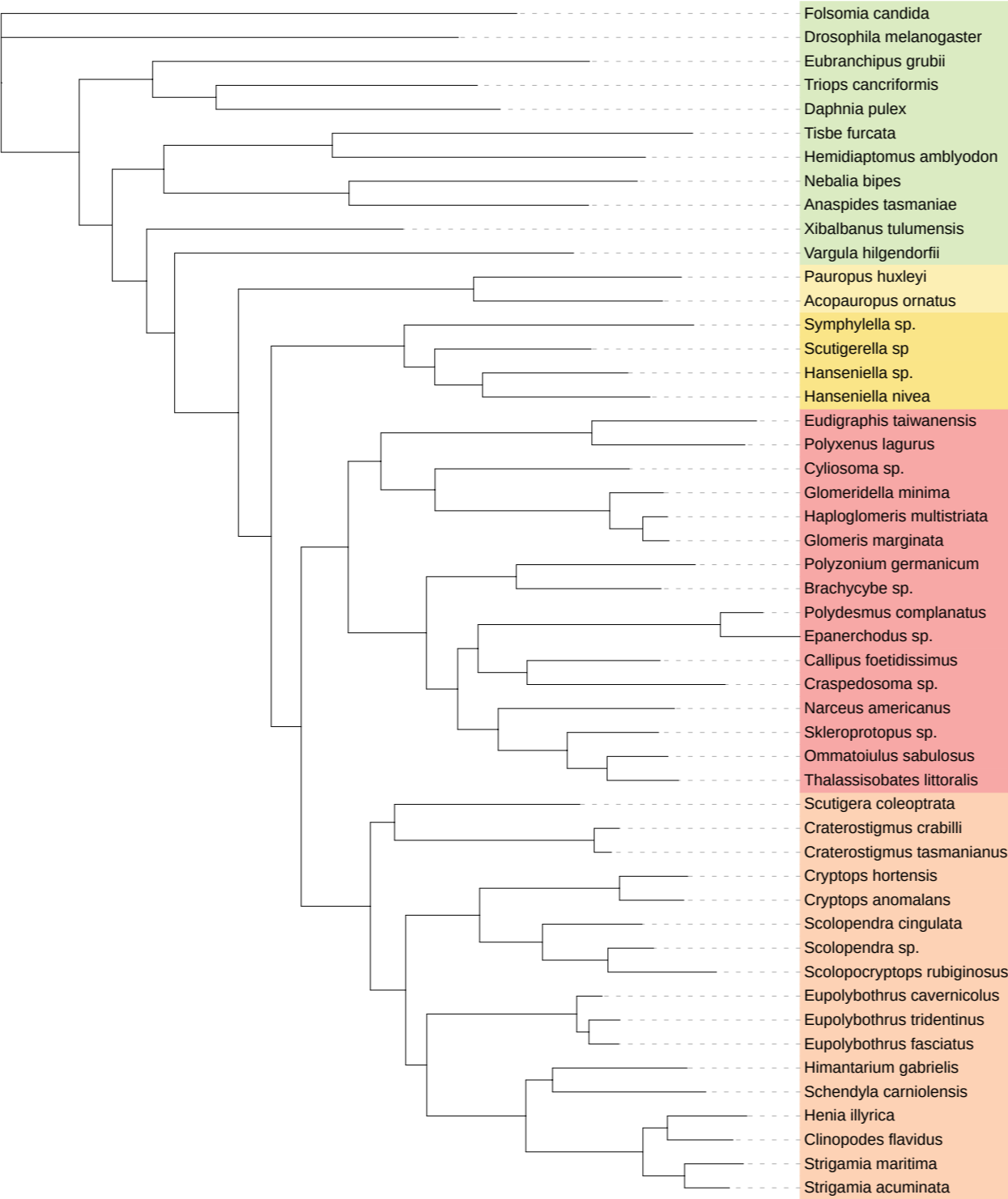

Supplement: Supplemental Information 6 [file peerj-09-12691-s006.pdf]

Tree scale: 0.1

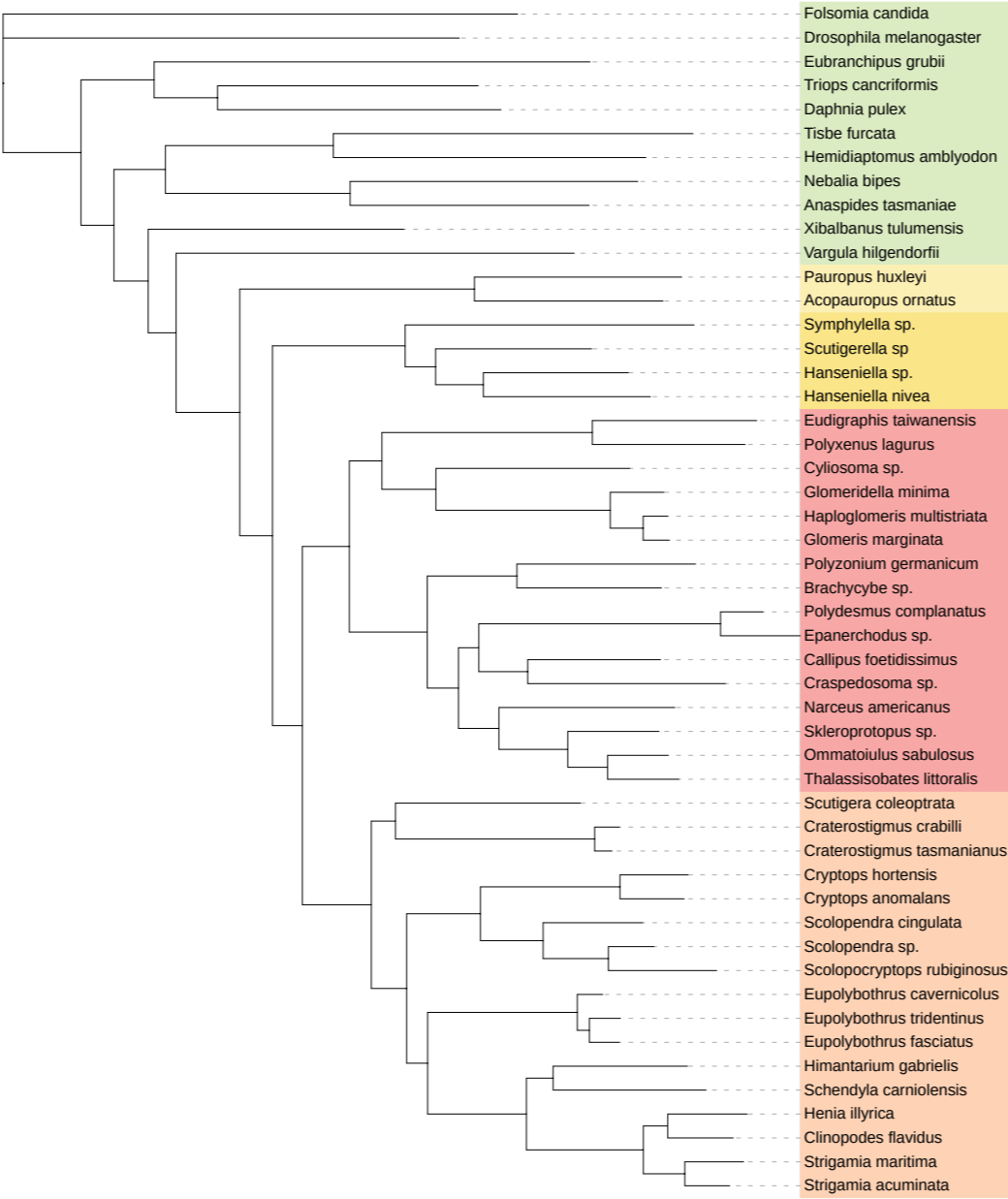

Supplement: Supplemental Information 7 [file peerj-09-12691-s007.pdf]
